# Assessing the functional coherence of modules found in multiple-evidence networks from *Arabidopsis*

**DOI:** 10.1186/1471-2105-12-203

**Published:** 2011-05-25

**Authors:** Artem Lysenko, Michael Defoin-Platel, Keywan Hassani-Pak, Jan Taubert, Charlie Hodgman, Christopher J Rawlings, Mansoor Saqi

**Affiliations:** 1 Centre for Mathematical and Computational Biology, Rothamsted Research, Harpenden, Herts, AL5, 2JQ, UK; 2 CPIB, Multidisciplinary Centre for Integrative Biology, School of Biosciences, University of Nottingham, Sutton Bonington, LE12 5RD, UK

## Abstract

**Background:**

Combining multiple evidence-types from different information sources has the potential to reveal new relationships in biological systems. The integrated information can be represented as a relationship network, and clustering the network can suggest possible functional modules. The value of such modules for gaining insight into the underlying biological processes depends on their functional coherence. The challenges that we wish to address are to define and quantify the functional coherence of modules in relationship networks, so that they can be used to infer function of as yet unannotated proteins, to discover previously unknown roles of proteins in diseases as well as for better understanding of the regulation and interrelationship between different elements of complex biological systems.

**Results:**

We have defined the functional coherence of modules with respect to the Gene Ontology (GO) by considering two complementary aspects: (i) the fragmentation of the GO functional categories into the different modules and (ii) the most representative functions of the modules. We have proposed a set of metrics to evaluate these two aspects and demonstrated their utility in *Arabidopsis thaliana*. We selected 2355 proteins for which experimentally established protein-protein interaction (PPI) data were available. From these we have constructed five relationship networks, four based on single types of data: PPI, co-expression, co-occurrence of protein names in scientific literature abstracts and sequence similarity and a fifth one combining these four evidence types. The ability of these networks to suggest biologically meaningful grouping of proteins was explored by applying Markov clustering and then by measuring the functional coherence of the clusters.

**Conclusions:**

Relationship networks integrating multiple evidence-types are biologically informative and allow more proteins to be assigned to a putative functional module. Using additional evidence types concentrates the functional annotations in a smaller number of modules without unduly compromising their consistency. These results indicate that integration of more data sources improves the ability to uncover functional association between proteins, both by allowing more proteins to be linked and producing a network where modular structure more closely reflects the hierarchy in the gene ontology.

## Background

The ever-increasing availability of high-volume proteomic, genomic and transcriptomics datasets has led to multiple studies aimed at the systems-level interpretation of this information using biological networks and relationship networks. Biological networks are graphs where the nodes are molecules and edges indicate interactions between them [1,2]. As explained in [1], in this type of network an allowance can be made for "suppression of detail", e.g. the intermediate components of some interactions may be omitted and instead represented by an edge. Most commonly this type of abstraction is used to represent gene regulation, where the DNA-protein interaction, transcription and translation are represented by just one edge between the regulator and its target protein. Relationship networks [3] are a superset of biological networks, where there is no longer a restriction that an edge must represent an actual real-life process that links the two molecules, but instead may indicate a shared property, such as two proteins having the same type of protein domain or being mentioned in the same publication.

The types of data used for construction of such networks include, but are not limited to, sequence similarity [4], shared sequence features [5,6], genetic interactions [6-10], gene co-expression [5,6,11-14], protein-protein interaction [5-7,11,15-18], domain interaction [19,20] and term co-occurrence in the scientific literature [3,5,10,11,21]. These types of information can be analysed independently or integrated together in order to encompass a wider range of biological mechanisms, provide additional evidence of association between entities in the network and connect disjoint parts of the network. In these studies, different techniques have been developed for the analysis of relationship networks, but they follow similar approaches: partitioning the network into modules, identifying the graph-theoretic properties of the network and relating these to biological function. In this work we have adopted a similar approach and have devised a set of metrics for quantifying the functional coherence of the modules in order to explore the effect of using multiple evidence-types in an integrated relationship network of *Arabidopsis thaliana *proteins.

Clustering approaches work by identifying densely interconnected areas within a network [2] and are commonly used to detect modular structure in graphs. In the context of biologically-relevant networks, these groups are often referred to as functional modules [2,7]. Functional modules in biological networks are groups of molecules that are more linked to the other members of the group than to non-members and have similar function [1]. The modular structure can be used to infer function of as yet unannotated proteins [16], to discover previously unknown roles of proteins in diseases [22] as well as for better understanding of the regulation and interrelationship between different elements of complex biological systems [12]. The function of a module is commonly identified from the annotation of its members with respect to the Gene Ontology (GO) [23].

GO consists of three separate categories - Biological Process, Molecular Function and Cellular Component, where each category consists of a controlled vocabulary of terms structured as a directed acyclic graph with qualified edges describing the semantic relationship between these terms. Each protein can be annotated with multiple GO terms and inherits the annotation of the parent terms and this makes it challenging to quantify and analyse the functional similarity between GO annotations. This has stimulated a number of studies that have explored these problems in detail. In particular, the importance of quantitative characterisation of GO term specificity (information content, IC) was demonstrated by [24] and based on this metric, several pair-wise quantitative measurements were developed that take into account the structure and properties of the Gene Ontology (reviewed in [25]). In a related set of efforts, a number of metrics were also designed to measure semantic consistency of protein sets. Original approaches were designed to identify the functional annotations for which a group of proteins was significantly enriched and did not take into account hierarchical structure of GO [26]. Although useful, these methods have a number of limitations, which were discussed in detail in the following studies [26,27]. To address these shortcomings, a number of extensions were proposed that combine some aspects of enrichment-based methods with adjustments for the relationship between the terms [28-30]. At the same time, another set of measures was also developed for the quantification of overall relatedness of annotations, rather than their overrepresentation within the set [27,31-34]. In this study we have drawn upon the insights that have emerged from this work in order to define a descriptive measure for comparison of functional annotation of protein sets.

We claim that to determine the biological relevance of the partitioning of a set of proteins there are two important aspects that need to be taken into consideration. The first is that the set of GO terms that best describes the common function of a representative proportion of proteins in the modules can be found at any annotation specificity level. However, at the higher levels, which are close to the root of the Gene Ontology, the annotation will not be particularly informative. This leads to a trade-off between the specificity of annotation terms and the number of proteins in the module to which it applies. The needs of the particular application case may dictate which of these two components is more important, and metrics have been developed that allow the emphasis to be placed on one or the other [35]. Using the metric defined in this paper (AIC-MICA), we were able to explore these two properties in five different relationship networks. The second aspect to be considered is that the sets of proteins with the same GO annotation can be fragmented, i.e. assigned to a number of different clusters by the clustering algorithm. Not only can the functionally similar group be spread across a number of clusters, but also may be more or less concentrated in the clusters where it is present.

To assess the functional coherence of modules from a relationship network both of these aspects, namely the representative functions of modules and the fragmentation of functional categories, are also relevant. Here we explore the potential of combined relationship networks to recover functional modules by considering four sources of information: protein-protein interaction (PPI), co-expression (COE), sequence similarity (SEQ) and co-occurrence of terms in the scientific literature (LIT). We have also constructed a combined network (ALL), which is a union of these four networks. These evidence types were chosen because they are often used for inferring functional relationships among genes and proteins and are readily available from the application of high throughput 'omics techniques. A large amount of co-expression data are available for *Arabidopsis thaliana *(see for example, [36]). Measurements of sequence similarity can be obtained for all pairs of proteins [37] and co-occurrence of protein terms in abstracts can be extracted from the scientific literature [38]. We decided to restrict the set of proteins in the network to those for which protein-protein interaction information is available, as we currently consider this to be limiting for *Arabidopsis*. This restriction means that we are only considering a small subset of *Arabidopsis *proteins, but has the advantage that it leads to a more balanced distribution of evidence types from the four information sources among the relationships between proteins. This setting allows us to evaluate to what extent patterns and trends that were previously found in whole proteome-based networks still hold in situations where only a subset of the whole proteome is analysed. Another motivation is to evaluate the usefulness of these approaches to extract the best possible information under conditions when data are scarce or incomplete.

As we have demonstrated in our previous work [39], by combining data from multiple resources it is possible to assemble much larger and more comprehensive integrated datasets. Several providers also offer pre-integrated datasets for *Arabidopsis*, such as STRING [18] and AtPIN [40]. However, the integration protocols of these data sources are not always clearly described even though the protocol can affect the structure of the network [39].

We have used the Ondex data-integration and visualization platform [41] to integrate and analyse the information sources and we have verified that the dataset used for this study is sufficiently representative by performing the same type of analysis on the data held in the STRING database for the same set of proteins.

## Results

### Network properties

The contributions from the four information sources to the edges in the network are shown in Table 1. There are 2355 proteins and 25172 links in the in the combined network - which is more than five times the number of links in the PPI dataset (4427). The links in the network exclusive to the possible evidence sources: co-expression (COE), co-occurrence of protein names (LIT), protein interaction (PPI) or sequence similarity (SEQ) are 36%, 18%, 14% and 25% respectively. The intersection of all evidence types is also very small (0.04%). This suggests that in this case each source of evidence tends to introduce new links into the combined network rather than reinforcing the relationships already found in other sources.

**Table 1:** Number of edges in the graph with evidence from the four information sources after applying a threshold on the relevant strength of the relationships (as defined in the Methods section)

COE	LIT	PPI	SEQ	Exclusive combinations		Inclusive combinations	
				N	%	N	%
✓	✓	✓	✓	9	0.04	9	0.04
✓	✓	✓		34	0.14	43	0.17
✓	✓		✓	83	0.33	92	0.37
✓	✓			84	0.33	210	0.83
✓		✓	✓	17	0.07	26	0.10
✓		✓		63	0.25	123	0.49
✓			✓	15	0.60	260	1.03
✓				9093	36.12	9534	37.88
	✓	✓	✓	123	0.49	132	0.52
	✓	✓		482	1.91	648	2.57
	✓		✓	692	2.75	907	3.60
	✓			4441	17.64	5948	23.63
		✓	✓	240	0.95	389	1.55
		✓		3459	13.74	4427	17.59
			✓	6201	24.63	7516	29.86

Exclusive combinations count edges where the evidence that links two nodes comes only from the sources indicated, while inclusive combinations allow the possibility of additional evidence types also supporting that link.

The global properties for the relationship networks constructed from the four constituent information sources and the combined network (ALL) are shown in Table 2. As expected, the combined network has fewer connected components since evidence from the other data sources connects previously unconnected nodes, and the size of the largest component is larger than that of any of the constituent networks. The diameters of the largest connected component of the SEQ, LIT and combined network (ALL) are of similar size (9, 9 and 10 respectively) and smaller than the COE and PPI networks (15 and 18 respectively), suggesting more cohesive or dense graphs. The increased density and the larger size of the main connected component indicate that the ALL network is likely to be much harder to optimally partition using a clustering approach. The transitivity gives a measure of clique-likeness of a graph and this is highest for the SEQ network (probably reflecting protein family structures) and the COE network possibly reflecting shared transcriptional regulatory mechanisms.

**Table 2:** A comparison of graph theoretic properties for the different evidence types

Evidence Network Type	Transitivity	Number of connected components	Size of the largest connected component	Diameter of the largest connected component
LIT	0.223	15	981	9

COE	0.580	24	991	15

PPI	0.070	100	1882	18

SEQ	0.746	268	241	9

ALL	0.406	9	2330	10

Since the initial dataset consisted of those proteins for which interaction data was available we would expect no unconnected proteins in the PPI and ALL networks. The number of orphan proteins (i.e. unconnected) for the SEQ, COE and LIT networks were 855, 1304 and 1343 respectively. The numbers of orphan proteins, however, depend on the score thresholds chosen (refer to Methods for the values used in this study).

### Network Clustering

**Figure 1:**
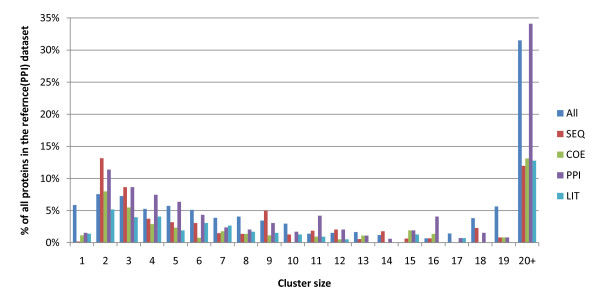
**Histogram of cluster size in combined (ALL), protein-protein interaction (PPI), co-occurrence of protein names (LIT), sequence similarity (SEQ) and co-expression (COE) networks**.

The four single information source networks and the combined network (ALL) were clustered using the MCL algorithm [42]. The distribution of cluster sizes is shown in Figure 1. The SEQ and PPI networks have a large number of clusters of size 2 and 3. The integrated network (ALL) and protein interaction network (PPI) contained the greatest number of larger clusters (size 20+). In the ALL network there are a large number of clusters of size 1. A total of 138 size 1 clusters contain 6.22% of all proteins in the network. This may be related to the cohesiveness of the ALL network, with tightly connected groupings leading to the exclusion of nodes by the MCL algorithm.

To explore the functional groupings of proteins in the network, we combined *Arabidopsis *GO annotations from three sources: IntAct [43], GOA-EBI [44] and UNIPROT [45]. Information content-based measures (see Methods section) were used to evaluate annotation specificity.

We wished to explore (i) whether the clusters contain proteins that are generally similar in terms of their functions, as assigned by Gene Ontology terms (the most representative GO terms in a cluster) (ii) the way in which proteins with the same functional roles are distributed across different clusters (the fragmentation of GO terms).

### Coverage and specificity of the most representative function of modules

The utility of clustering depends on being able to group together a large enough number of proteins, so as to facilitate exploring the modular structure of the network without diluting the information content of the clusters to such an extent that the groupings do not capture biologically meaningful relationships.

The Average Information Content of the sets of these Most Informative Common Ancestor GO terms (AIC-MICA) was used to determine the coverage and the specificity of the most representative function of modules (AIC-MICA is defined in the Methods). If a cluster contained proteins that were of very diverse function, we would expect that the GO categories corresponding to the most representative functions would not be very specific, i.e. the Most Informative Common Ancestor (MICA, see [24]) would be close to the root of the Ontology tree and thus would not represent a functionally meaningful grouping. Given that the links in a relationship network may not always reflect accurate functional relationships, we do not look for the MICA of all the proteins in the cluster. Instead we measure the Average Information Content (AIC) associated with a set of MICA of at least a certain coverage (percentage of all proteins in a cluster), sampled at 10% increments from 40% to 90%. This method allows simultaneous detection of functional similarities in more than one functional category and is more robust to outliers - as only a certain proportion of the proteins in the cluster need to share functional similarity in order for their ancestor GO term to be included in the set.

**Figure 2:**
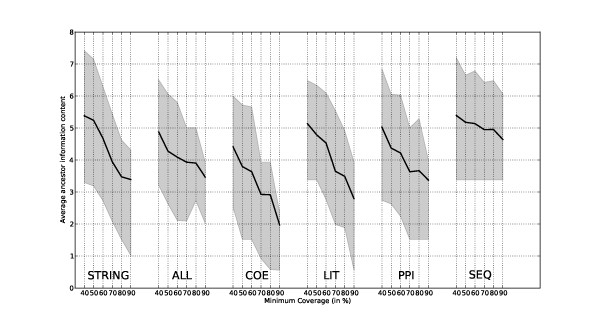
**The Average Information Content across clusters of the Most Informative Common Ancestor (AIC-MICA) requiring that the information content measure includes 40% to 90% of the proteins in a cluster**. The solid line is the average IC and the shaded areas are 25 and 75 percent quartiles.

In Figure 2 the AIC-MICA is plotted for the five relationship networks constructed for this study as well as for the weighted version of the STRING database. As expected, the average information content of the representative GO terms decreases with the increase in cluster coverage, namely the requirement that the common ancestor includes a greater proportion of proteins in the cluster. Average information content in the LIT network is similar to the ALL at lower coverage range (40%-50%), but declines very sharply and is second worst at the higher coverage level. This may be an indication that although useful associations can be found using term co-occurrence, these clusters tend to be less coherent at the whole-cluster level. Clusters in the COE network have the lowest information content at all coverage levels. The information content at coverage level of 90%, (namely the information content of most informative common ancestor that includes 90% of the proteins in a cluster) is highest for the SEQ network followed by the ALL network. In the SEQ network, however, only 1496 proteins are assigned to clusters (of size greater than 1) whereas in the ALL network this figure is 2217. For proteins that cannot be assigned to a module this means that no inference can be made using the guilt-by-association principle. So, for only 5.9% of proteins, no insight can be gained from clustering the ALL network, whereas for the SEQ network this figure is 36.5%. Therefore, the ALL network has a much greater potential for suggesting biological context, supporting our hypothesis that the integration of multiple information sources can be beneficial when identifying functional modules.

The STRING comparison analysis was undertaken using a complete set of information from the STRING database for the same set of 2355 proteins. The results indicate that the performance of STRING at the higher coverage (80-90%) levels was comparable to that of the ALL network. We have also considered individual evidence types from STRING (coexpression, literature and experimental PPI detection), which were found to be similar to the results obtained for the corresponding datasets constructed for this paper, if the data are interpreted as an un-weighted network. The results of this analysis can be found in the Additional File 1: additional figures and analysis.

### Modules in the ALL relationship network and their most representative functions

**Figure 3:**
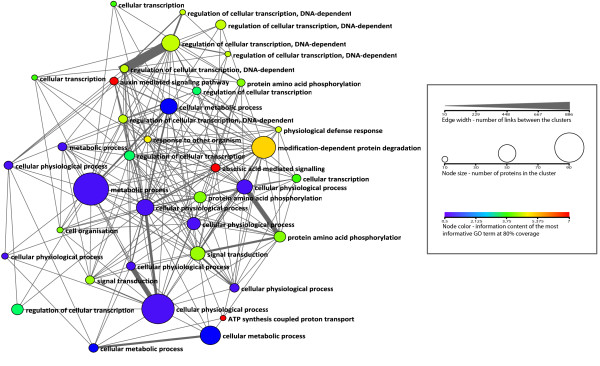
**Modular structure of the combined network of all evidence types**. Nodes show all clusters with 10 or more members with edges indicating the number of links between them. All clusters are annotated with the most informative GO term at 80% of the clusters proteins annotated with the GO term.

Figure 3 shows the modular structure found in the ALL network produced by application of the MCL algorithm. The clusters are annotated with the most informative of the representative GO terms at 80% level. This high-level view illustrates the meta-structure of the relationship network as it was resolved by the clustering algorithm. The overview shows that, although the network is very densely interconnected, the clustering algorithm has performed reasonably well, with only a few cases where a very large number of links were made between two clusters. One example of where the clustering was not optimal is where two clusters with the same annotation "regulation of cellular transcription, DNA-dependent" were linked together by more than 800 edges, but were not brought together. In total there were 6 of the 36 clusters with this same annotation, even though the annotation level is quite specific, where information content lies in the middle of the range (4.0 - coloured green). Interestingly, this phenomenon was also seen in other annotations relating to signalling and regulation of transcription. The two clusters with the most informative annotation are both related to hormone signalling (coloured red). There is also one large cluster annotated to "modification-dependant protein degradation", a similar cluster related to protein catabolism was also found in other studies that analysed PPI and co-expression networks [16,46].

We have explored the possibility of further post-processing the clusters produced by the MCL algorithm by looking at the average FSWeight [47,48] of the pairs of clusters; but the results proved inconclusive. Further information about this analysis is included in the Additional File 1: additional figures and analysis. However, we did observe that the average FSWeight of edges inside clusters was significantly higher than that for edges connecting different clusters.

### Fragmentation of functional categories

The other aspect that needs to be taken into consideration when assessing the functional coherence of modules is the fragmentation of functional categories. Here, we examined how the Gene Ontology terms were distributed across the clusters.

Clustering can result in proteins with the same functional annotation being split across multiple clusters. This leads to the separation of this group of proteins into multiple fragments. In Table 3, the Best Fragment Rank Proportion (BFRP) indicates that the sets of proteins annotated with the same GO term are least fragmented in the ALL network. This suggests that the combined network performed better than the individual networks for grouping together entities with similar GO annotation. To evaluate the level of fragmentation of functional categories, both the number of fragments and their size distribution need to be considered. The entropy of the fragmentation (refer to Methods for definitions) gives us a measure of this size distribution. As can be seen in Table 3, the Best Entropy Rank Proportion (BERP) is also maximal for the ALL network, followed by the LIT network, indicating that overall the entropy with respect to GO categorisation is also lowest in these networks.

**Table 3:** The first two rows show the average entropy for the networks and, for comparison, the average entropy for the networks with GO labels randomly permuted

	ALL	SEQ	COE	PPI	LIT
**Average entropy (actual network)**	2.72	2.96	3.30	2.86	2.96

**Average entropy (randomly permuted network)**	3.31	3.43	3.42	3.29	3.33

**Relative decrease in entropy (compared to randomly permuted network)**	17.8%	13.7%	3.5%	13.1%	11.1%

**BFRP**	49.43%	22.78%	3.55%	24.56%	28.43%

**BERP**	39.58%	16.64%	2.75%	18.58%	31.18%

The third row details the decrease in entropy observed in the actual network compared to the randomly permuted network. By both methods of measuring network fragmentation, the ALL dataset produces the best quality networks. The percentages may not add up to a 100%, as in cases where several networks performed the same all were counted as "best" for that GO term. The fourth and fifth rows show the best fragment rank percentage and best entropy rank percentage statistics (defined in the Methods section).

A lower entropy value implies more ordered data, both in terms of reduced fragmentation and prevalence of larger fragments. Table 3 shows the average entropy values for each network and for the corresponding control networks where cluster labels have been randomly permuted for all GO categories. To avoid the problems of small sample sizes, only those GO categories that were assigned to at least 10 proteins in the dataset were included. The ALL network has the lowest average entropy, again suggesting that it is better at grouping together related proteins, the average entropy being 2.72 compared with 3.31 for the same networks with GO labels associated with the nodes, being randomly permuted. All of the observed differences in entropy were found to be highly significant, with none of the permuted networks having an average entropy value greater than that of the real one, indicating a confidence of at least p < 0.0001. This appears to be due to the hierarchical nature of the GO categories, where every wrong assignment with respect to a child term would also lead to penalties incurred at the parent level. Therefore, the density distributions for the permuted networks were very narrow (Plot is included in the Additional File 1: additional figures and analysis).

### An example of fragmentation in the ALL relationship network

**Figure 4:**
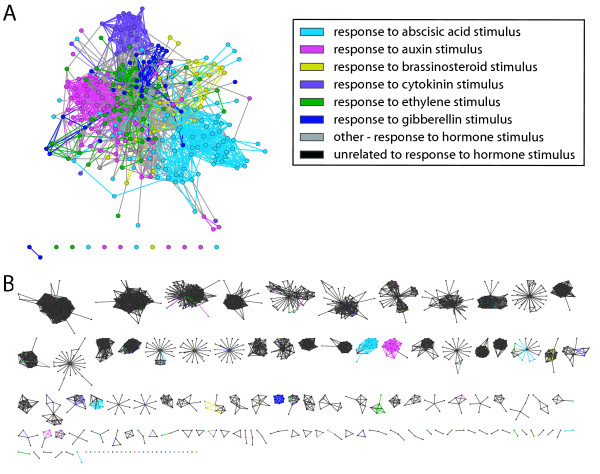
**A subnetwork from the combined (ALL) network of proteins annotated with the GO term "response to hormone stimulus"**. The diagram shows (A) the proteins annotated to this GO term and direct links between them and (B) the breakdown of this group of proteins into clusters. The colouring is consistent between the two panels. Proteins that are not annotated to this process are hidden on panel (A) and are coloured grey in panel (B). In (B) all clusters shown contain at least one member with "response to hormone stimulus" annotation and the only edges shown are the ones that link two members of the same cluster.

Figure 4A shows all proteins (nodes) in the combined (ALL) network annotated to the high level GO term "response to hormone stimulus" and its more specialised categories (grey clusters). The average shortest path length (SPL) between all proteins with this annotation was 20% shorter compared to a control, where node labels were permuted 10000 times. The SPL reduction in distance for the child terms listed in Figure 4 was even greater and ranged from 22-30%. It is interesting to note that the SPL distance between child terms echoes the hierarchy of the Gene Ontology, which was defined entirely independently by manual curation.

Figure 4B shows the fragmentation of this cluster by visually separating all the MCL clusters across which this term is distributed. It is evident that the clustering in this case is not able to group together all the nodes that are associated with the general process 'response to hormone stimulus". In this case there were only two clusters (of size greater than 10) that have most proteins in the cluster annotated with the same term (e.g. 'response to auxin stimulus' and 'response to abscisic acid stimulus'). However, even in the situations when the grouping is suboptimal, it is still useful to be able to determine how much the grouping differs from the one specified by annotations and structure of the Gene Ontology.

## Discussion

In order to assess the functional coherence of modules detected by clustering relationship networks combining four commonly used data sources we have looked at the representative functions of these modules with respect to GO categories and at the fragmentation of GO categories with respect to the modules. To investigate the trade-off between coverage and specificity of the representative function of modules, we have defined the AIC-MICA metric. Additionally, two metrics describing the fragmentation of GO categories, namely BFRP and BERP, were introduced to evaluate how well the modular structure recovered by the MCL algorithm corresponds to the BP categories. These metrics look at two key aspects that relate modules in relationship networks to functional annotations. They allow us to compare the usefulness of individual data sources and the effects of combining multiple sources on the coherence of the modules.

We have found that, as expected, the SEQ network was the best for recovering very specific functional association between proteins. This was evident from the high AIC-MICA values across all coverage levels. However, an important point to note is that it may not always be desirable to extract such close groupings, and the higher level categorisation may be helpful to provide a broad overview or to help dissect very large datasets. Compared to other networks, SEQ consisted of a large number of strongly connected components (results not shown) which resulted in the relatively high overall entropy with respect to the whole Gene Ontology. We also observed that the clusters recovered were only related to a small number of GO terms. Another problem with SEQ as a sole data source is that there was insufficient evidence to link most of the proteins in our reference set. By comparison with the SEQ network, it was possible to use the ALL network to assign 721 more proteins to a cluster of size greater than one due to links that were contributed by other sources. Based on these findings, we conclude that overall there is a clear benefit from the integration of additional data sources, although there is a small cost incurred because of a reduction in functional coherence. As the ALL network performs relatively well in terms of AIC-MICA (40-90), this dilution of annotation specificity does not appear to render it uninformative. In fact, the minimum information content value that was applicable at a 40% coverage level was 0.55 and was reached only for 5 clusters found in the ALL network. This value corresponds to the 'cellular physiological process' GO term, which is one of the direct descendants of the 'biological process' root term, and is therefore very general.

To support this work, several different visualisation strategies were developed that help to summarise complex integrated networks and identify high-level patterns in them. Using these visualisation methods, we have identified that there was a hierarchically organised neighbourhood in the integrated network that was composed of the proteins annotated to the "response to hormone stimulus" GO term. This finding indicates there may be more complex and meaningful patterns than just the modules that could be identified using clustering approaches.

Comparison of graph-theoretic properties of the four networks also appears to indicate that the addition of extra edges lead to the creation of a more compact network, with smaller diameter than the COE or PPI networks. Despite this, the transitivity has remained relatively low - indicating that the number of complete cliques is small. These differences may be interpreted as an indication that, in the ALL network, potential modules are more difficult to recover and the results may be further improved using more robust clustering approaches, like spectral clustering methods [49]. Further investigation of the impact of increasing complexity of the network versus increasing levels of noise that arise from integration of additional data sources is necessary to confirm these trends.

The co-expression (COE) network performed the worst with respect to BFRP, BERP and AIC-MICA. At first glance, this result appears to contradict several earlier studies [12,13] where many meaningful clusters were identified in the co-expression network but this discrepancy is likely to be an artefact of the smaller subset of the proteome that was used in this case. In earlier reports using large co-expression networks, the patterns detected tended to be associated with clusters containing more than a 1000 proteins [12,13], which are much larger than any of the modules identified in this study. This may be an indication that co-expression is a weaker source of evidence of functional similarity and more data are necessary in order to be able to make useful inferences.

In this study we have restricted the set of proteins in the network to those for which protein-protein interaction information is available, as this is a currently limiting information source for *Arabidopsis*. Using a larger set of proteins would have meant that the contribution of the PPI data would have been highly unbalanced in relation to other available information. Although we recognise that there are other species, in particular *Saccharomyces cerevisiae*, for which there is much more data available, it is also of importance to validate these types of approaches in more complex multicellular model organisms. We have also illustrated that meaningful modules can be successfully identified by clustering the integrated relationship networks even in situations when limited data are available and only part of the complete proteome is considered.

In this work we have addressed a number of important issues pertinent to the identification of functional modules in integrated relationship networks, but it is important to recognise that a number of alternative approaches exist for analysis of such networks. In particular, it is possible to weight the edges in the network based on the confidence in individual evidence types [47,48,50-53]. However, both the strategies of selecting optimum weights and the ways they can be meaningfully combined across heterogeneous evidence types still remains a subject of ongoing research. Another possibility is to use an alternative clustering approach for the recovery of modules. Historically, the MCL algorithm has often been applied in the context of biological networks because it offers scalable performance even with large datasets and several studies have shown that it can outperform other methods in some cases [54-56]. However, a number of other novel algorithms have now been developed, among them MCODE [57], MC-UPGMA [58] CPA [59] FORCE [60] and SPICi [61]. A number of these approaches have also been compared in the context of PPI networks in the work by Brohée and van Helden [62]. Further investigation into these alternative approaches has potential for future research, but was outside the scope of the present study.

## Conclusions

Module detection in integrated biological and relationship networks is one of the most important tools for interpretation of complex biological datasets. As the amount of biological information continues to grow, it also becomes increasingly important to improve our understanding of inter-relationships within these data and, ultimately, their relationship to biological function. In this paper we have explored and quantified the integration of the several data types that are most commonly used for construction of such networks. For our datasets, we have found that combining several types of evidence was beneficial with respect to the functional annotation of modules detected using MCL clustering algorithm, that on average more closely corresponded to the functional groupings in the Biological Process aspect of GO. Although the overall level of informativeness of cluster annotation was not as good as in the sequence similarity network, it was possible to link many more proteins using additional information sources. These findings indicate that there is benefit to the integration of additional information sources, as it allows more proteins to be assigned to functional modules with only a relatively small reduction in the module annotation precision. The overall outcomes of this study provide a number of insights into the relationship between integrated networks and protein function and may be of use for further refinement of related approaches that can better capture biologically relevant information from integrated datasets.

## Methods

We constructed a protein-protein interaction network based on experimentally established protein-protein interaction data from the IntAct database [43] and combined it with additional data, namely gene co-expression, sequence similarity and information from co-occurrence of protein names in the scientific literature. Previously we have described the approach used for constructing a combined network of PPI and gene co-expression data using Ondex [39]. In this study we investigate the inherent modular structure of these networks and relate it to the underlying biological processes using the Gene Ontology (GO) [23] and quantify these properties using information content and semantic distance-based measures.

### Construction of the integrated relationship network

In the network, nodes represented proteins and edges were added if there was at least one of the possible four evidence types linking these two proteins: co-occurrence of protein names in PubMed abstracts, co-expression of genes that encode those proteins (where the magnitude of the Pearson correlation coefficient is greater than 0.6), sequence similarity (with E-value < 0.0001) or experimentally determined protein-protein interaction.

We have imported protein-protein interaction (PPI) data from IntAct database (PSI-MI XML format) into the Ondex system and removed all entities that were not annotated with *Arabidopsis thaliana *NCBI taxonomy identifier and all entities that were not proteins. Then the interactions between multiple copies of the same protein were also discarded. All proteins that were not part of any interactions were also removed from the set.

A CO-Expression network (COE) was constructed from *Arabidopsis *co-expression data from the ATTED-II [63,64] database. An edge was created in the co-expression network if the absolute value of Pearson's correlation coefficient of respective gene expression profiles was greater than 0.6.

For the literature-based co-occurrence analysis of protein names, we downloaded 30,639 abstracts from PubMed which contained the word "*Arabidopsis*". This set of publications together with the integrated set of *Arabidopsis *PPIs were loaded into Ondex. Each protein node contained a complete set of protein names and synonyms provided by TAIR and UNIPROT. The Ondex text mining plug-in was used to create relations between proteins and publications and transform the output to a co-occurrence network [38]. An edge in the protein name co-occurrence network (LIT) indicates that there was at least one abstract that included a mention of both proteins.

Sequence similarity was determined by using TimeLogic^® ^Tera-BLAST™ (Active Motif Inc., Carlsbad, CA) for all-against-all sequence-comparison of proteins in the interaction dataset, with an E-value cut-off at 10^-3 ^and minimum percent sequence identity cut-off at 25%. One edge was created in a sequence-similarity network (SEQ) per pair of proteins with similar sequences.

### Gene Ontology annotation

To explore the functional groupings of proteins in the network, we have combined all available *Arabidopsis *GO annotations from three sources: IntAct [43], GOA-EBI [44] and UNIPROT [45]. We have calculated the Information Content (IC) [65] of the annotations using the combined set of all GO annotations of the *Arabidopsis *proteome subset as identified in the UNIPROT database. All annotations to proteins not included in the proteome set were discarded prior to calculation of the IC. The combined network of different evidence types and GO annotation is included in the additional material (Additional File 2: Integrated network).

### Clustering the relationship networks

We explored the natural groupings of the proteins (nodes) using the MCL clustering algorithm [42]. This algorithm simulates flow in the network and can be used to identify strongly connected groups of nodes in the network. We have used an implementation of MCL (v10-148) algorithm from http://www.micans.org/mcl/, which was wrapped as a plug-in and made accessible from the Ondex data integration platform. The inflation coefficient (I) determines the granularity of the clusters produced by the algorithm. A value of I = 2.8 was used for all of the clustering analysis described in this paper.

### Assessing the functional coherence of modules

Our aim was to assess the functional coherence of modules by exploring two aspects (i) whether the clusters contain proteins that are generally similar in terms of their functions, as assigned by Gene Ontology terms, i.e. the most representative GO terms in a cluster (ii) the way in which proteins with the same functional roles are distributed across different clusters, i.e. the fragmentation of the GO terms.

**Figure 5:**
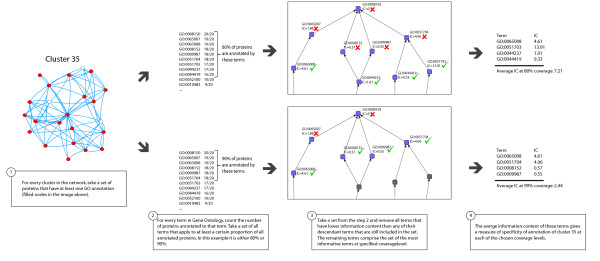
**Example calculation of the average information content for cluster coverage level**.

To study the first aspect of the functional coherence, we have developed a measure that quantifies the annotation similarity at various levels of coverage. Since the GO is described by a DAG, one way of estimating the overall level of commonality of GO terms in a cluster is to find a set of representative common ancestors. Each of these ancestors will depend on how many of the total number of proteins in the cluster have some GO annotations we chose to include. For clusters in the ALL, PPI, LIT, COE, SEQ networks, we have found a set of representative common ancestors corresponding to 40-90% of the terms, and have calculated the associated IC of this ancestor term. The approach is illustrated schematically in Figure 5. The statistic calculated is an Average Information Content of the Most Informative Common Ancestor set (AIC-MICA).

In order to study the second aspect of the functional coherence, we have introduced two metrics to evaluate the fragmentation of the GO annotation terms in the different clusters. Given a term *t*, from the biological process aspect of GO, a set *A_t _*of all proteins annotated to the term *t*, we define *N_t _*the number of fragments of *t *as the cardinality of the set of clusters *C_t _*that contain at least one element of *A_t_*. We also define *p_k_*, the proportion of the total number of proteins annotated with term *t *that are found in cluster *k*:

with *k *∈ *C_t_*.

**Figure 6:**
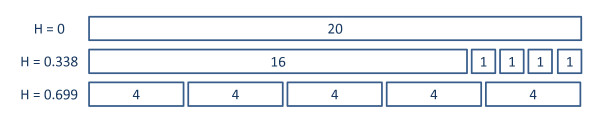
**Schematic diagram showing how entropy provides a useful metric of fragmentation of a given GO term across clusters**. If 20 proteins are associated with a given GO term and they all are in the same cluster then the entropy (H) is zero. If most (16) of the proteins are in one cluster and the remaining proteins are in separate clusters H = 0.338. However, as the proteins get more evenly distributed across clusters the entropy increases.

And the entropy (*H_t_*) as:

Similarly to the number of fragments *N_t_*, the entropy *H_t _*gives us a measure of the fragmentation of the term *t *across the clusters but it also accounts for the distribution of the size of the fragments (see Figure 6). To ensure that the variations in entropy observed between the different clusters were statistically significant, the average entropy for each of the real networks was compared to a randomized control by permuting cluster labels 10000 times. The analysis described in the paper can be reproduced by following the protocol provided (Additional File 3: Instructions for reproducing the analysis). This can be done by loading and executing the workflow (Additional File 4: Ondex analysis workflow) using Ondex software.

In order to compare the number of fragments and the entropy of fragmentation according to the source of relationship data, we have ranked both of them for each of the GO terms across all five networks. The number of times each of the data sources were assigned the best rank (i.e. the lowest value) was counted and a proportion with respect to the total number of GO categories was calculated. For the sake of brevity from here onwards, we use abbreviations BFRP (best fragment rank proportion) and BERP (best entropy rank proportion) when referring to these comparative measures. These measures provide an intuitive method to compare the networks, as the output can be understood as a percentage of cases where a particular network performed best or at least as good as one of the others.

### Visualisation

The integration process was implemented as a set of workflows in the Ondex integrator [66]. The resulting network was visualized and further analyzed in an interactive manner using the Ondex front-end. Ondex contains a command console that supports a variety of common scripting languages and allows the use of external libraries to facilitate the analysis, add additional annotation to nodes and edges and then visualize the results. To carry out the analysis for this paper, we have chosen to use Jython in order to be able to utilize the analysis capabilities offered as part of the NetworkX v0.99 Python graph analysis library [67]. To enable exchange of data between Ondex and NetworkX, we have implemented a method that allows export of a pre-defined subset of an integrated network for NetworkX representation, the results returned were then added as additional annotation to the graph using methods, in Ondex Jython scripting plug-in. Interactive visual exploration of the network used the visualization methods available in Ondex, which include an ability to set the visibility, size/width and colour of nodes and edges based on the numerical values of their attributes and/or group membership.

## Authors' contributions

AL implemented the methods described in the paper, performed the analyses and prepared most of the figures. KHP contributed a method for determining term co-occurrence from the scientific literature and assisted with preparing figures. JT assisted with the data integration platform Ondex. MS, MDP, CJR supervised the project and provided input in the design. All authors were involved in the preparation of the manuscript. CH is AL's academic supervisor and assisted in the preparation of the manuscript. All authors read and approved the manuscript.

## Supplementary Material

Additional file 1 **Additional figures and supplementary analyses**. This file contains a figure showing the entropy distributions of the permuted networks, FSWeight analysis results, and a figure presenting the results of AIC-MICA application to different subsets of the STRING database. Click here for file

Additional file 2 **Integrated network in Ondex file format**. Integrated network, which was used to perform the analysis for this paper. Can be viewed using Ondex frontend, available at: http://sourceforge.net/projects/ondex/files/supp/BMC_Lysenko_2011/ONDEX.zip/download Click here for file

Additional file 3 **Instructions for reproducing the analysis**. Description of how to deploy the Ondex software and use it to re-produce the AIC-MICA analysis. Click here for file

Additional file 4 **Ondex analysis workflow in Ondex workflow file format**. The Ondex workflow file for running the AIC-MICA analysis. Can executed using the Ondex Integrator tool, available as part of Ondex frontend suite: http://sourceforge.net/projects/ondex/files/supp/BMC_Lysenko_2011/ONDEX.zip/download Click here for file
