# Supplementary material for: Assessing the functional coherence of modules found in multiple-evidence networks from Arabidopsis
Source: BMC Bioinformatics. 2011 May 25;12:203. doi: 10.1186/1471-2105-12-203 (PMC3118170; doi:10.1186/1471-2105-12-203)
Supplement: Additional file 1 — Additional figures and supplementary analyses. This file contains a figure showing the entropy distributions of the permuted networks, FSWeight analysis results, and a figure presenting the results of AIC-MICA application to different subsets of the STRING database. [file 1471-2105-12-203-S1.DOC]

**2 Application of the FSWeight for the post-processing of the MCL clusters**

To explore the possibility of identifying and resolving the sub-optimal MCL clusters shown on the Figure 3 of this paper we have explored the possibility of using the FSWeight metric to score the edges. The FSWeight metric was calculated on the ALL network, using the following formula :


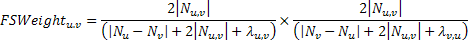


Where
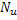
 and
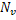
 are the sets of direct neighbours of the nodes
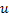
 and
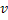
,
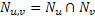
 and
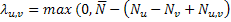
 . We have found that there is a highly significant (t (17691) = 48.39, p < .001) difference in the average FSWeight of the edges connecting nodes inside of the clusters compared to the average weight of the edges connecting the members of the different clusters:

Then, we have considered all pairs of clusters with more than 5 members and have looked at the potential increase in the AIC-MICA(70) score if the clusters were to be combined, calculated according to the following formula:

This score was then compared to the average FSWeight of all the edges connecting this pair of clusters. We report that no strong correlation between the two was observed in this dataset:

However, as both Arabidopsis annotation sets and the PPI data are still very sparse and advocate for further evaluation of FSWeight in this setting using a more well-characterised species, like *Saccharomyces cerevisiae*.

1. Wong L: **Constructing More Reliable Protein-Protein Interaction Maps**. In: *International Symposium on Computational Biology & Bioinformatics.* 2008: 284-297.

2. Chua HN, Sung WK, Wong L: **Exploiting indirect neighbours and topological weight to predict protein function from protein-protein interactions**. *Bioinformatics* 2006, **22**(13):1623-1630.

**3 Application of the AIC-MICA analysis to STRING data**

## STRING data was analysed according to the same protocol described in the “****Clustering the relationship networks****”, “****Gene Ontology annotation****” and “Assessing the functional coherence of modules” section of the paper. The dataset was restricted to the same set of proteins as the one found in the ALL network found in the paper. The coexpression, experimental protein-protein interaction, literature and the combined network of all three were analysed, as well as the combined dataset of all evidence types used by STRING. We have analysed both weighted (using the weights provided by STRING) and the unweighted interpretations of these networks. It was found that the pattern of change in the AIC-MICA score at different coverage levels was very similar to the one found for the networks constructed for this paper. The weighted version appeared to perform better than an unweighted one. However, the performance of the ALL network at higher coverage levels of 80-90% was comparable to that of complete weighted STRING network (at the same coverage levels).
