# Supplementary material for: Assessing the functional coherence of modules found in multiple-evidence networks from Arabidopsis
Source: BMC Bioinformatics. 2011 May 25;12:203. doi: 10.1186/1471-2105-12-203 (PMC3118170; doi:10.1186/1471-2105-12-203)
Supplement: Additional file 3 — Instructions for reproducing the analysis. Description of how to deploy the Ondex software and use it to re-produce the AIC-MICA analysis. [file 1471-2105-12-203-S3.DOC]

**Instructions to reproduce the AIC-MICA analysis and source code availability**

Additional data includes five:

1. A source data file, containing integrated data in Ondex format (Additional file 2 : Integrated network)
2. An Ondex workflow file that was used to generate results, described in the paper(Additional File 4)
3. A file with instructions (this document – Additional File 3)

A pre-compiled version of the Ondex software used for this paper is available at: <ftp://ftp.rothamsted.bbsrc.ac.uk/downloads/longterm/Lysenko2011/ondex.zip>

Java v6 or compatible JVM must is needed to run the software. All source code for the software and the analysis described in the paper is available on SourceForge and can be openly accessed using the URL:

<http://ondex.svn.sourceforge.net/svnroot/ondex/branches/Aug09/>

For simplicity, a compiled version of the application used for analysis was also provided.

To run the analysis:

1. Uncompress the application distributive (ondex.zip) on a computer with sufficient system resources – at least 1.3 Gb of RAM must be available for Java in order to run the analysis.
2. Download and install an MCL clustering implementation from [www.micans.org/mcl/](../../../../D:%5Cprojects%5CDILS-remix%5Cadditional%20data%5Cwww.micans.org%5Cmcl%5C), according to the instructions of the developers
3. Start the application using runme.bat (on Windows) or runme.sh (on Linux)
4. From the top menu select File->Launcher 2.0 (beta) to start the workflow management tool
5. In the workflow tool window select File->Open and choose the workflow file
6. The analysis described in the paper is implemented as a batch plug-in called Analysis_dilsr. Once the workflow is loaded, modify the paths to file to represent the locations on your system – e.g. “InputFile” of Oxl plug-in, “MclDir” and “IndexDir” of each of the 5 Analysis_dilsr plug-in instances. “IndexDir” is location where some temporary files used for the analysis will be stored and can be any not write protected empty directory.
7. Run the workflow
8. Once the task is finished there will be output files produces in the main application directory. The AIC-MICA stats are in the files with like ”[I=2.8][COE=100.0]clusters_annotations.tab”. The columns in the file provide the following information: % term target – target coverage level of AIC-MICA, % term - actual coverage level, Name – name of the GO term that satisfies these conditions, IC – information content of the term, Cluster AIC-MICA – the statistics for every combination of Cluster id and % term target combination.
9. The network file will also be created called “BMC_set_after.xml.gz”, which contains the modified version of the dataset that includes annotations from the analysis. It can be loaded in the Ondex frontend by selecting File->Open from file from the main application menu.
10. All other analysis described in the paper was done using the standard, previously published and documented methods of the Ondex system. Instructions about how to use them are included in the form of help and tutorial files distributed with the main application. Combined with the methods description in the paper, this documentation provides all the information necessary to reproduce the integration of the datasets. Troubleshooting guides and examples are also available from the project website ([*http://www.ondex.org/*](http://www.ondex.org/)).
